# Non-Specific Binding, a Limitation of the Immunofluorescence Method to Study Macrophages In Situ

**DOI:** 10.3390/genes12050649

**Published:** 2021-04-27

**Authors:** Emma Sicherre, Anne-Laure Favier, Diane Riccobono, Krisztina Nikovics

**Affiliations:** 1 Imagery Unit, Department of Platforms and Technology Research, French Armed Forces Biomedical Research Institute, 91223 Brétigny-sur-Orge, France; emma.sicherre@supbiotech.fr (E.S.); anne-laure.favier@intradef.gouv.fr (A.-L.F.); 2 Radiobiology Unit, Department of NRBC Defence, French Armed Forces Biomedical Research Institute, 91223 Brétigny-sur-Orge, France; diane.riccobono@intradef.gouv.fr

**Keywords:** macrophages, in situ hybridization, hybridization-chain-reaction

## Abstract

Advances in understanding tissue regenerative mechanisms require the characterization of in vivo macrophages as those play a fundamental role in this process. This characterization can be approached using the immuno-fluorescence method with widely studied and used pan-markers such as CD206 protein. This work investigated CD206 expression in an irradiated-muscle pig model using three different antibodies. Surprisingly, the expression pattern during immunodetection differed depending on the antibody origin and could give some false results. False results are rarely described in the literature, but this information is essential for scientists who need to characterize macrophages. In this context, we showed that in situ hybridization coupled with hybridization-chain-reaction detection (HCR) is an excellent alternative method to detect macrophages in situ.

## 1. Introduction

The characterization of macrophages is essential in the fight against various diseases [1,2,3,4,5]. Many macrophage markers have been used for diagnostics or research for a long time [1,2,3,4]. Most investigations still use immunofluorescence method techniques for the detection of marker proteins in situ [6,7,8]. Despite the fact that this technique is very useful and well reproducible, there are some disadvantages, such as non-specific binding of the antibody to target the antigen. Nevertheless, non-specific binding is rarely described in scientific publications focusing on in situ studies.

In contrast, in situ hybridization (HIS) is a more specific method to study macrophages in situ despite being more fastidious and cumbersome [9,10,11]. In HIS, the mRNA involved in the protein translation is used to analyze the gene expression. The probe (cRNA or cDNA complementary to the mRNA) used during in situ hybridization is not only specific, but also suitable to distinguish between protein variants [12]. It also broke free from commercial antibodies targeting specific animal models. Among the non-radioactive approaches, the most widely used techniques are the digoxigenin (DIG) technique, with DIG labeled probes, and the fluorescence in situ hybridization (FISH), with various molecules incorporated into the probes (DIG, Biotin, Dinitrophenyl, Fluorescein) [13,14]. The first one is very sensitive but allows only the study of a single gene while several genes can be considered within the same sample with the second one, despite a relatively lower sensitivity. Recently, a new method was developed—in situ hybridization coupled with hybridization-chain-reaction detection (HCR) [15,16]. The advantage of this approach is to combine multiple labeling with a higher sensitivity. 

In the present work, the in-situ expression of CD206 marker was studied in a regenerating irradiated pig muscle after adipose tissue stem cell treatment (IR-ASC) [17,18,19]. Three different antibodies, described by suppliers as able to detect CD206 marker, however, showed a different pattern, and two of them produced significant amounts of false-positive signals. The percentage of the CD206-expressing cells in IR-ASC muscle differed for each antibody. A double analysis was performed to compare in situ hybridization (in situ-DIG and in situ-HCR) and immunofluorescence methods. Results showed that in situ hybridization approaches were much more suitable than immunofluorescence for the in-situ quantification of the CD206 marker and, by extension, to study macrophages in situ.

## 2. Materials and Methods

### 2.1. Experimental Design

As previously described, minipigs were locally irradiated in the lumbar area with a 60Cobalt source with a dose rate of 0.6 Gy/min until reaching a dose of 50 Gy at the entry area. The minipigs were randomly separated into two groups, after local irradiation. Three minipigs obtained intramuscular injections of ASC (25 × 106 ASC) 25, 46 and 66 days after irradiation [17].

### 2.2. Blocking Reagents

To block non-specific bindings, slides were incubated with 3% Bovine Serum Albumin (Eurobio, GAUBSA01, Paris, France) in phosphate-buffered saline pH 7.4 (PBS), a ready-to-use antibody diluent (Diagomics, ZUC025-100, Blagnac, France), 2% donkey serum in PBS (Abcam, ab7475, Cambridge, UK), SmartBlock of Blocking Sampler Package Small (Diagomics, 113050, Blagnac, France) and a ready-to-use Emerald antibody diluent (Sigma, 936B-08, Lyon, France).

### 2.3. Immunofluorescence

Irradiated and ASC-treated muscles were harvested, immersed in liquid nitrogen and stored at −80 °C. From the frozen samples, sections were made using a cryostat microtome (10 μm) (Cryostat FSE Shandon, Thermo Electron Corporation, United States). Sections were fixed in 4% formaldehyde in PBS. Cells were permeabilized on slides for 15 min with 0.5% Triton X100 (*v*/*v*) buffered with PBS. After three washes with PBS, non-specific binding sites were blocked with Emerald antibody diluent (Sigma 936B-08) for one hour. The sections were then incubated overnight at 4 °C with the primary rabbit Anti-CD206 (1) (Abcam, ab64693) at 1:100 dilutions, the primary mouse Anti-CD206 (2) (Santa cruz Bio. sc-376108 (D1)) at 1:100 dilution, or the primary goat Anti-CD206 (3) (Santa cruz Bio. sc-34577 (C20)) at 1:500 dilution in Emerald antibody diluent. Sections were washed for 20 min in PBS and incubated with either the secondary donkey anti-rabbit Alexa Fluor 488 conjugate (Thermo Scientific, A-21206) at 1:500 dilution, the secondary donkey anti-mouse Alexa Fluor 568 conjugate (Thermo Scientific, A10037) at 1:500 dilution, or the secondary donkey anti-goat Alexa Fluor 647 conjugate (Abcam, ab150131) at 1:500 dilution for two hours at room temperature and rinsed. Finally, sections were washed in PBS for 20 min and mounted using a Fluoroshield mounting medium with DAPI (Abcam, ab104139). The fluorescence was detected using an epifluorescence microscope DM6000 (Leica, Germany) equipped with monochrome and color digital cameras and 470 ± 40 nm, 527 ± 30 nm, 630 ± 75 nm, 700 ± 75 nm filters (Leica, 11504203, 11504165, 11504207, 11504171, respectively).

### 2.4. In Situ Hybridization

In situ hybridization methods were performed as described [19]. Oligos designed for in situ hybridization experiments were listed in Table S1.

### 2.5. Microspectrofluorimetry

Emission fluorescence spectra was recorded with a Zeiss LSM800 confocal microscope with lasers 488, 561 and 640 nm. Anti-CD206 (1) / Alexa Fluor 488 (turquoise fluorescence) was imaged with 488 nm excitation. Emission spectra was measured between 460 and 720 nm (bandwidth 5 nm). Anti-CD206 (2)/Alexa Fluor 568 (red fluorescence) was imaged with 568 nm excitation. Emission spectra was measured between 560 and 720 nm (bandwidth 5 nm). Anti-CD206 (3)/Alexa Fluor 647 (yellow fluorescence) was imaged with 488 nm excitation. Emission spectra was measured between 620 and 720 nm (bandwidth 5 nm).

### 2.6. Statistical Analysis

Statistical analyses were performed with an ANOVA-Tukey test [20].

### 2.7. Homology Sequence Analysis

Homology sequence analysis was performed with Blastp program on using CD206 pig (NP_001242898) and human (CAH71176) protein sequences and sus scrofa entire genome (taxid:9823). 

## 3. Results

### 3.1. Identification of CD206-Expressing Cells in the IR-ASC Muscle by the Immunofluorescence Method

**Figure 1:**
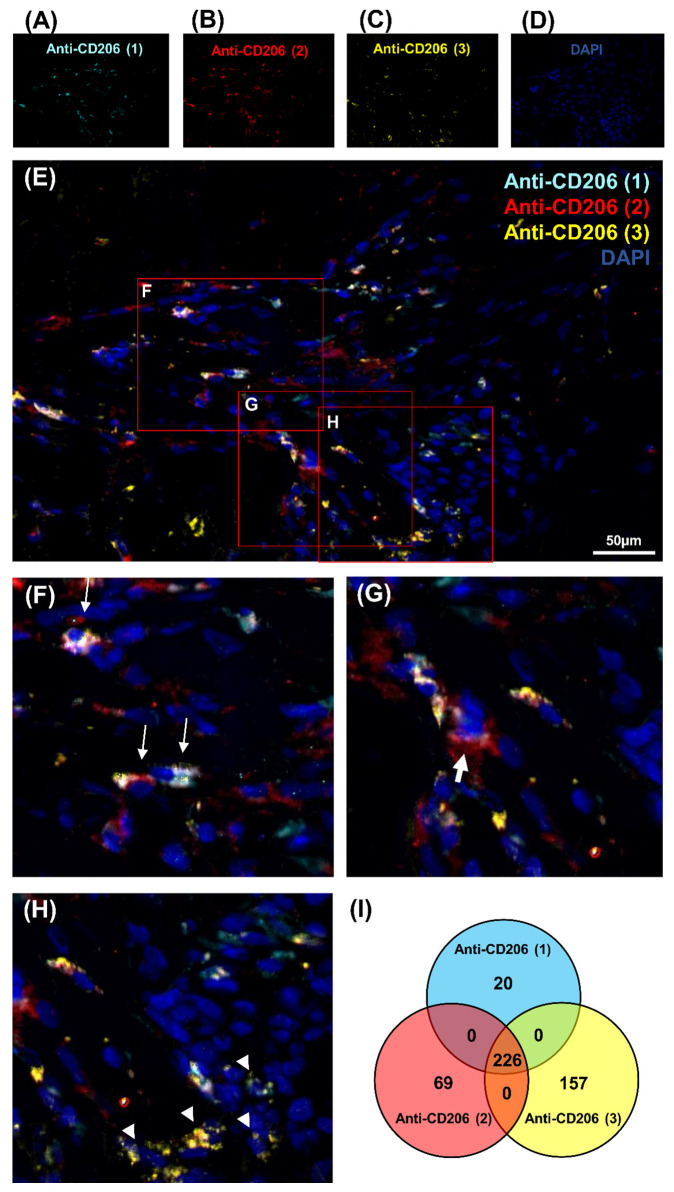
Detection of CD206 marker expression in cells in IR-ASC muscle by immunofluorescence method. (**A**) Anti-CD206 (1) (Alexa488, turquoise fluorescence), (**B**) Anti-CD206 (2) (Alexa488, red fluorescence), (**C**) Anti-CD206 (3) (Alexa488, yellow fluorescence) labeling, (**D**) Nuclear staining with DAPI (blue fluorescence). (**E**) Merged image, (**F**–**H**) expanded view: high magnification image of the area within the red rectangles in image E. (**I**) Venn diagram of CD206-positive cells of 3000 cells. Thin arrow: cells labeled with three antibodies; thick arrow: cells labeled with Anti-CD206 (2) antibody only; arrowhead: cells labeled with Anti-CD206 (3) antibody only.

This study focused on CD206 expression in regenerating muscle to understand the macrophage polarization during regeneration. The first attempt to study CD68 expression gave unsuccessful results with only a weak signal that could be related to the low homology of 68% between *sus scrofa* and *homo sapiens* CD68 sequences (Figure S1A,B). Consequently, three different antibodies, Anti-CD206 (1), Anti-CD206 (2), Anti-CD206 (3), were used in a pig model, as protein homology raised 88.53% (Figure S2). Moreover, no cross-reactivity was obtained between the CD206 pig protein sequence and *sus scrofa* using BLASTp. Several blocking reagents were tested, Emerald antibody dilution was selected because of the poor level of non-specific interactions observed. Despite the fact that theoretically all three antibodies should recognize the same protein, the three distinct antibodies exhibited different patterns (Figure 1A–E). Significant variability was detected in CD206 expression in IR-ASC muscle. To demonstrate differences in cell labeling, three representative zones were selected (Figure 1F–H). Some cells showed a positive signal with all three antibodies—Anti-CD206 (1, 2, 3)^+^—being more visible at a higher magnification (Figure 1F). Indeed, some cells were labeled only with one antibody—Anti-CD206 (2)^+^ (Figure 1G) or Anti-CD206 (3)^+^ (Figure 1H). 

**Figure 2:**
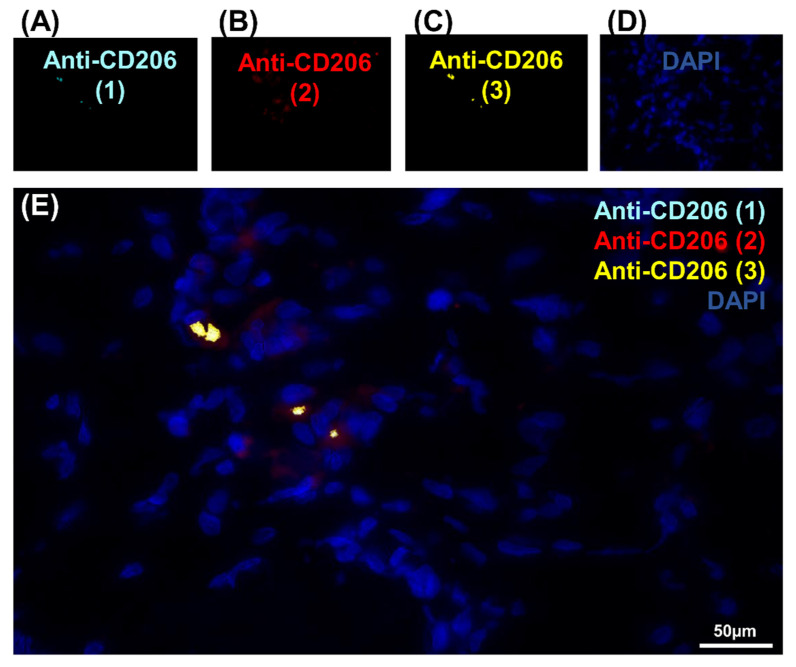
Negative controls of the CD206 marker expression in cells in IR-ASC muscle by immuno-fluorescence method. (**A**) Negative control of Figure 1A with Anti-CD206 (1) (Alexa488, turquoise fluorescence). (**B**) Negative control of Figure 1B with Anti-CD206 (2) labeling (Alexa488, red fluorescence). (**C**) Negative control of Figure 1C with Anti-CD206 (3) labeling (Alexa488, yellow fluorescence) labeling. (**D**) Negative control of Figure 1D with nuclear staining with DAPI (blue fluorescence). (**E**) Merged image.

To pursue our investigation, quantification of CD206-expressing cells was performed in order to better understand the correlation between these populations of cells (Figure 1I, Figure S3 and Table 1). Quantitative analysis was based on random examination of three sets of 1000 cells for each condition (Figure S3). Both Anti-CD206 (2) and Anti-CD206 (3) showed a significant number of positive cells, respectively (Figure S3), and was illustrated by a diagram Venn (Figure 1I). As resumed in Table 1, the ratio between CD206-expressing cells differed: 8.2% for Anti-CD206 (1)^+^, 9.83% for Anti-CD206 (2)^+^ and 12.76% for Anti-CD206 (3)^+^; the Anti-CD206 (1,2,3)^+^, estimated at 75.3 ± 7.8 per 1000, served as a reference in the pairwise comparisons to calculate the p-value. No double-positive cells for antibody pairs 1–3, 2–3, 1–2 suggested that all true CD206^+^ cells were recognized. No labeling was observed with any of the antibodies in the negative control (absence of primary antibody) (Figure 2A–E).

### 3.2. Identification of CD206-Expressing Cells by Microspectrofluorimetry

**Figure 3:**
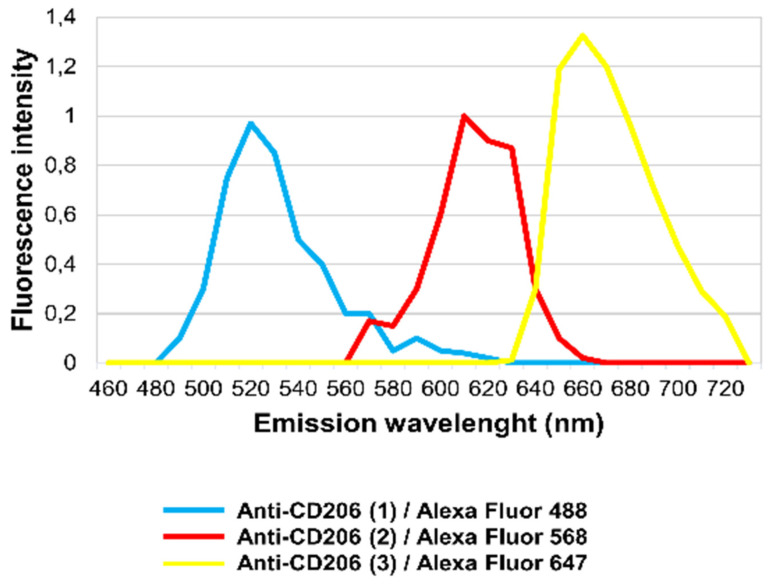
In situ fluorescence microspectroscopy analysis of the Anti-CD206 (1, 2, 3) antibodies in IR-ASC muscle. Emission spectra of Anti-CD206 (1)/Alexa Fluor 488 (turquoise line), Anti-CD206 (2)/Alexa Fluor 568 (red line) and Anti-CD206 (3)/Alexa Fluor 647 (yellow line).

It has long been known that different structural proteins and also certain de novo produced molecules (Flavin-type molecules) may yield strong autofluorescence in tissues [21,22]. Diverse autofluorescence signals may be differentiated using their specific emission spectra [23]. To demonstrate that the labeling (Anti-CD206 (2)^+^ and Anti-CD206 (3)^+^) was due to unspecific antibody binding and was not the result of autofluorescence, in situ microspectroscopical analysis was performed. Each immunofluorescence labeling could be separated based on its own emission spectra (Figure 3). Anti-CD206 (1), Anti-CD206 (2) and Anti-CD206 (3) antibodies were recognized by a secondary antibody conjugated to Alexa Fluor 488, Alexa Fluor 568 and Alexa Fluor 674, respectively. The emission spectrum of Anti-CD206 (1)/Alexa Fluor 488 fluorescence reached a maximum at 520 nm (turquoise line; Figure 3) while the emission spectrum of Anti-CD206 (2)/Alexa Fluor 568 fluorescence and Anti-CD206 (3)/Alexa Fluor 647 peaked at 610 nm and 660 nm, respectively (red and yellow lines; Figure 3). 

In situ, the detected fluorescence in IR-ASC muscle was altogether specific to Alexa Fluor 488, Alexa Fluor 568 and Alexa Fluor 647 molecules, demonstrating that there was no autofluorescence. 

### 3.3. Identification of CD206-Expressing Cells in the IR-ASC Muscle by In Situ Hybridization

**Figure 4:**
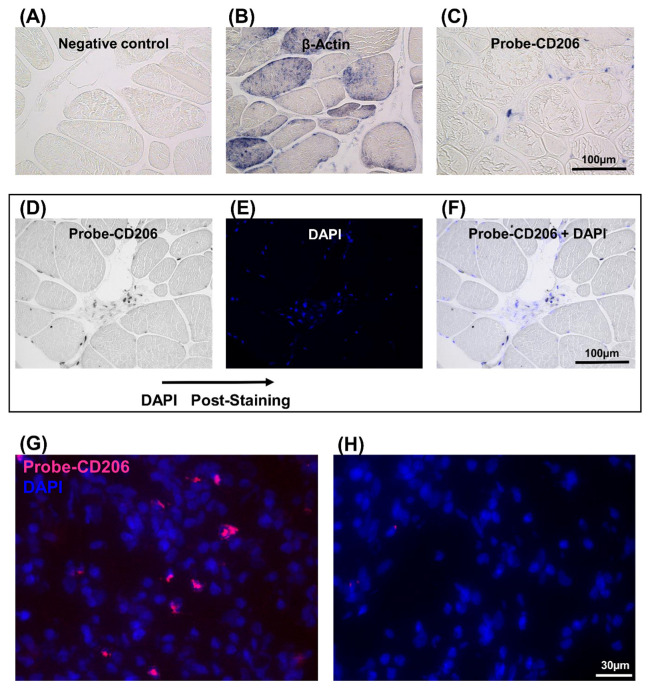
Detection of CD206 expression in the IR-ASC muscle by in situ hybridization. (**A**–**F**) In situ-DIG; (**G**,**H**) In situ-HCR. (**A**) Negative control. (**B**) Expression of β-actin mRNA (positive control). (**C**) Expression of CD206 mRNA. (**D**–**F**) For the quantification, slide was post-stained with DAPI. (**D**) Expression of CD206 mRNA. (**E**) Nuclear staining with DAPI (blue fluorescence). (**F**) Both CD206 mRNA and DAPI staining. (**G**) Expression of CD206 mRNA. (**H**) Negative control of G. Probe-CD206 (Alexa546, red fluorescence), nuclear staining with DAPI (blue fluorescence).

Since the immunofluorescence method did not provide an unambiguous response for CD206 gene expression, further investigations were undertaken using in situ hybridization. Therefore, the expression of CD206 was analyzed by two different in situ hybridization methods in the regenerating muscle (Figure 4). In the first, an in situ-DIG technique was employed. A digoxigenin-labeled cRNA probe, corresponding the CD206 mRNA, was generated. This DIG-probe was hybridized with the CD206 gene mRNA and a specific antibody detected DIG molecules. In the absence of the specific probe, no expression was observed (Figure 4A). The expression of β-actin was used as a positive control (Figure 4B). CD206 expression was well-detectable in IR+ASC muscle (Figure 4C) compared to the nontreated muscle (Figure S4). The number of CD206-expressing cells (Figure 4D) and post-stained nucleus with 4’,6-diamidino-2-phénylindole (DAPI) were quantified (Figure 4E). The merged image (Figure 4F) was used to determine the ratio of CD206^+^ positive cells (Table 1). This experiment indicated that approximately 6.7% of cells were CD206^+^.

In the second, an in situ-HCR technique was carried out to detect CD206 expression in the IR+ASC muscle (Figure 4G). The advantage of this technique is that it uses different fluorophore-labeled DNA hairpins for the detection of multiple mRNAs [15,16,24]. A random examination of 1000 cells in the IR-ASC muscles indicated that 7.2% of the cells expressed CD206 mRNA (Table 1). No expression was detected in the absence of specific probe (Figure 4H).

## 4. Discussion

Macrophages play an essential role in tissue regeneration [25,26,27]. The proper characterization of macrophages is essential for a better understanding of regenerative processes [3,28,29,30]. CD206 protein is a mannose receptor primary expressed on the surface of M2, M2-like and tissue specific macrophages [31,32,33]. In the present study, we analyzed the expression of CD206 marker by immunofluorescence method, microspectrofluorimetry and two in situ hybridization techniques (in situ-DIG and in situ–HCR) in order to better characterize macrophages in situ. We showed that the detection of CD206 gene expression by the immunofluorescence method was not reliable because expression patterns differed depending on the antibody used. Therefore, the choice of blocking reagent and primary antibody is crucial to ensure the success of immunofluorescence detection. 

In situ-based methods appear more suitable for identifying the macrophage markers of different animal tissue origins and determining the number of CD206-expressing cells. Both techniques used showed a similar amount of CD206-positive cells with Anti-CD206 (1) antibody suggesting that this antibody is the only one that can be used safely for further work in contrast to the two other ones. 

## 5. Conclusions

In summary, (i) an appropriate blocking reagent could improve antibody labeling for immunofluorescence method; (ii) fluorescence spectromicroscopy analyses can be an advantage to separate the signal of interest from the tissue autofluorescence; (iii) the three specific antibodies against CD206 used to detect macrophages have specificity issues, and ISH/HCR is one potential alternative to localize macrophages in situ.

## Figures and Tables

**Table 1:** Quantitative analysis of CD206-expressing cells by immunofluorescence method and in situ hybridization. Random examination of three sets of 1000 cells per condition.

	Percentage of Cells Expressing CD206	P (ANOVA-Tukey Test) CD206 (1,2,3)^+^ as Reference
Anti-CD206 (1)	8.2	Not significant
Anti-CD206 (2)	9.83	<0.05
Anti-CD206 (3)	12.76	<0.05
In situ-DIG CD206	6.7	Not significant
In situ-HCR CD206	7.2	Not significant

## Supplementary Materials

The following are available online at https://www.mdpi.com/article/10.3390/genes12050649/s1, Figure S1. Detection of CD 68 marker expression in cells in IR ASC muscle by immunofluorescence method and homology sequence analysis between pig and human CD 68 protein sequences. Figure S2. Homology sequence analysis of pig and human CD206 proteins. Figure S3. Quantitative analysis of the CD 206 expressing cells, based on random examination of 3 sets of 1000 cells per condition. Figure S4. Expression of CD206 mRNA in the non-treated muscle by in situ DIG hybridization. Table S1. Oligos used for in situ hybridization experiments.
