# Supplementary material for: Non-Specific Binding, a Limitation of the Immunofluorescence Method to Study Macrophages In Situ
Source: Genes (Basel). 2021 Apr 27;12(5):649. doi: 10.3390/genes12050649 (PMC8145419; doi:10.3390/genes12050649)
Supplement: Supplementary file 1 [file genes-12-00649-s001.zip › FigureS1new.pdf]

(A)

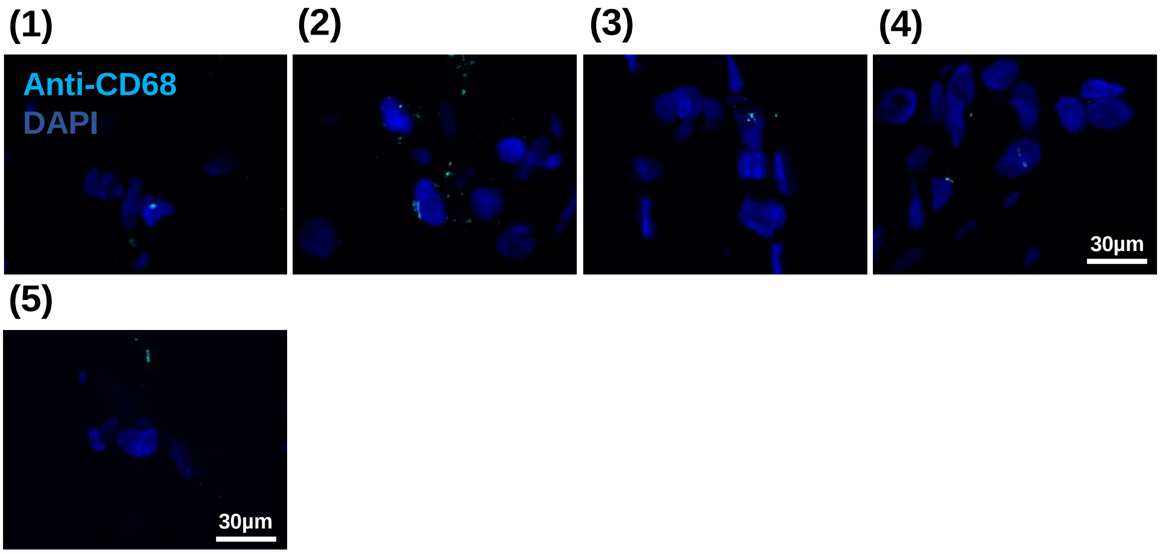

(B)

Pig: ***Sus scrofa*** **CD68 (XP_013834029)**
Human: ***Homo sapiens*** **CD68 (NP_001242)**

```
Pig    64   MRLAVIFSGALLGLLAAQDTGNECPHKKSATLLPSFTVTPTATESTGTPT------ATES  117
            MRLAV+FSGALLGLLAAQ TGN+CPHKKSATLLPSFTVTPT TESTGT +         +
Human  1    MRLAVLFSGALLGLLAAQGTGNDCPHKKSATLLPSFTVTPTVTESTGTTSHRTTKSHKTT  60

Pig    118  TGTPTATESTGTPTATESTASPETTRHRSTTQRTTTAGTPSHKSTTATHNPATATSH---  174
            T   T T +T     T +     T+    T   T+ +   S   +TATH+PAT TSH
Human  61   THRTTTTGTTSHGPTTATHNPTTTSHGNVTVHPTSNSTATSQGPSTATHSPAT-TSHGNA  119

Pig    175  TVHPTSNSTATSPESSTGSPHPGPPPPTPSPSPCSKEVKGDYTWTNGSQPCVHLQAQIQI  234
            TVHPTSNSTATSP   T S HP PPPP+PSPSP SKE  GDYTWTNGSQPCVHLQAQIQI
Human  120  TVHPTSNSTATSP-GFTSSAHPEPPPPSPSPSPTSKETIGDYTWTNGSQPCVHLQAQIQI  178

Pig    235  RVLYPTQGGGKAWGISVVNPNKTKVQGGCEGAHAHLLLSFPYGQLSFGFKQEPLKSTVYL  294
            RV+Y TQGGG+AWGISV+NPNKTKVQG CEGAH HLLLSFPYG LSFGF Q+  +  VYL
Human  179  RVMYTTQGGGEAWGISVLNPNKTKVQGSCEGAHPHLLLSFPYGHLSFGFMQDLQQKVVYL  238

Pig    295  NYMAIEHNVSFPGAVQWTFSVQNSSLRELQTPLGQSYSCRNASIILSPAFHLDLLSLKLQ  354
            +YMA+E+NVSFP A QWTFS QN+SLR+LQ PLGQS+SC N+SIILSPA HLDLLSL+LQ
Human  239  SYMAVEYNVSFPHAAQWTFSAQNASLRDLQAPLGQSFSCSNSSIILSPAVHLDLLSLRLQ  298

Pig    355  AAQLPPTGAFG  365
            AAQLP TG FG
Human  299  AAQLPHTGVFG  309
```

Figure S1. (A) Detection of CD68 marker expression in cells in IR-ASC muscle by immunofluorescence method. (1) Anti-CD68 (Bethyl Lab., A500-018A mouse, 1:500; Alexa488, turquoise fluorescence), (2) Anti-CD68 (Thermo Fisher, MA5-13324 mouse, 1:200; Alexa488, turquoise fluorescence), (3) Anti-CD68 (Abcam, ab955 mouse, 1:100; Alexa488, turquoise fluorescence), (4) Anti-CD68 (Abcam, ab125212 rabbit 1:1,000; Alexa488, turquoise fluorescence) labeling. (5) Negative control of A,B,C,D. Nuclear staining with DAPI (blue fluorescence). (B) Homology sequence analysis between pig and human CD68 protein sequences.
