# Supplementary material for: Non-Specific Binding, a Limitation of the Immunofluorescence Method to Study Macrophages In Situ
Source: Genes (Basel). 2021 Apr 27;12(5):649. doi: 10.3390/genes12050649 (PMC8145419; doi:10.3390/genes12050649)
Supplement: Supplementary file 1 [file genes-12-00649-s001.zip › FigureS2new.pdf]

Pig: *Sus scrofa* CD206 (NP_001242898)
Human: *Homo sapiens* CD206 (CAH71176)

```
Pig    1     MRLSPCLAFLSFLPVALQLLDTRQFLIYNEDHKRCVEALSPSSVQTAVCNQDNEAQKFRW  60
             MRL   L F S +P A+ LLDTRQFLIYNEDHKRCV+A+SPS+VQTA CNQD E+QKFRW
Human  1     MRLPLLLVFASVIPGAVLLLDTRQFLIYNEDHKRCVDAVSPSAVQTAACNQDAESQKFRW  60

Pig    61    VSESQIMSVAFKLCLGVPSKTDWVPVTLYACDSKSEFQKWECRNDTLLGIKGEDIFFNYG  120
             VSESQIMSVAFKLCLGVPSKTDWV +TLYACDSKSEFQKWEC+NDTLLGIKGED+FFNYG
Human  61    VSESQIMSVAFKLCLGVPSKTDWVAITLYACDSKSEFQKWECKNDTLLGIKGEDLFFNYG  120

Pig    121   NRQEKNIMLYKGSGLWSRWKVYGTTDDLCSRGYEAMYTLLGNSNGATCAFPFKFENKWYA  180
             NRQEKNIMLYKGSGLWSRWK+YGTTD+LCSRGYEAMYTLLGN+NGATCAFPFKFENKWYA
Human  121   NRQEKNIMLYKGSGLWSRWKIYGTTDNLCSRGYEAMYTLLGNANGATCAFPFKFENKWYA  180

Pig    181   DCTTAGRSDGWLWCGTTTDYDTDKLFGYCPLKFEGIERLWNKDPLTSISYQINSKSALTW  240
             DCT+AGRSDGWLWCGTTTDYDTDKLFGYCPLKFEG E LWNKDPLTS+SYQINSKSALTW
Human  181   DCTSAGRSDGWLWCGTTTDYDTDKLFGYCPLKFEGSESLWNKDPLTSVSYQINSKSALTW  240

Pig    241   HQARKSCQQQNAELLSITEIHEQTYLTGLTSSLTSGLWIGLNSLSFNSGWQWSGGSPFRY  300
             HQARKSCQQQNAELLSITEIHEQTYLTGLTSSLTSGLWIGLNSLSFNSGWQWS  SPFRY
Human  241   HQARKSCQQQNAELLSITEIHEQTYLTGLTSSLTSGLWIGLNSLSFNSGWQWSDRSPFRY  300

Pig    301   LNWLPGSPSAEPGKSCVSLNPGKNAKWENLQCVQKLGYICKKGNTTLNSFVIPSESDVPT  360
             LNWLPGSPSAEPGKSCVSLNPGKNAKWENL+CVQKLGYICKKGNTTLNSFVIPSESDVPT
Human  301   LNWLPGSPSAEPGKSCVSLNPGKNAKWENLECVQKLGYICKKGNTTLNSFVIPSESDVPT  360

Pig    361   SCPSQWWPYAGHCYKIYREEKKIQRDALTACRKEGGDLASIHSIEEFDFIISQLGYEPND  420
              CPSQWWPYAGHCYKI+R+EKKIQRDALT CRKEG DLASIH+IEEFDFIISQLGYEPND
Human  361   HCPSQWWPYAGHCYKIHRDEKKIQRDALTTCRKEGSDLASIHTIEEFDFIISQLGYEPND  420

Pig    421   ELWIGLNDIKIQMYFEWSDGTPVTFTKWLPGEPSHENNRQEDCAVMKGKDGYWADRACER  480
             ELWIGLNDIKIQMYFEWSDGTPVTFTKWL GEPSHENNRQEDC VMKGKDGYWADR CE
Human  421   ELWIGLNDIKIQMYFEWSDGTPVTFTKWLRGEPSHENNRQEDCVVMKGKDGYWADRGCEW  480

Pig    481   PLGYICKMKSQAQTPGRVEVETGCRKGWKRHGFYCYLIGHTLSTFAEANQTCENEKAYLT  540
             PLGYICKMKS++Q P  VEVE GCRKGWK+H FYCY+IGHTLSTFAEANQTC NE AYLT
Human  481   PLGYICKMKSRSQGPEIVEVEKGCRKGWKKHHFYCYMIGHTLSTFAEANQTCNNENAYLT  540

Pig    541   TVEDRYEQAFLTSLVGLRPERYFWTGLSDVQNKGTFQWTIAEGVQFTHWNTDMPGRKAGC  600
             T+EDRYEQAFLTS VGLRPE+YFWTGLSD+Q KGTFQWTI E V+FTHWN+DMPGRK GC
Human  541   TIEDRYEQAFLTSFVGLRPEKYFWTGLSDIQTKGTFQWTIEEEVRFTHWNSDMPGRKPGC  600

Pig    601   VAMRTGVAGGLWDVLRCEEKNKFVCKHWAEGVTRPPEPTTTPEPKCPEDWGTSTKTSLCF  660
             VAMRTG+AGGLWDVL+C+EK KFVCKHWAEGVT PP+PTTTPEPKCPEDWG S++TSLCF
Human  601   VAMRTGIAGGLWDVLKCDEKAKFVCKHWAEGVTHPPKPTTTPEPKCPEDWGASSRTSLCF  660

Pig    661   KLFAKGKHEKKTWFESRDFCRALGGDLASINNKEEQQAIWRLVTASGSYHELFWLGLTYS  720
             KL+AKGKHEKKTWFESRDFCRALGGDLASINNKEEQQ IWRL+TASGSYH+LFWLGLTY
Human  661   KLYAKGKHEKKTWFESRDFCRALGGDLASINNKEEQQTIWRLITASGSYHKLFWLGLTYG  720

Pig    721   SPSEGFTWSDGSPVSYENWAYGEPNNYQNVEYCGELKSDAGMSWNDINCEHLNNWICQIR  780
             SPSEGFTWSDGSPVSYENWAYGEPNNYQNVEYCGELK D  MSWNDINCEHLNNWICQI+
Human  721   SPSEGFTWSDGSPVSYENWAYGEPNNYQNVEYCGELKGDPTMSWNDINCEHLNNWICQIQ  780

Pig    781   KGQTPKPEPTPAPQDNPPVTEDGWVIYKDYQYYFSKEKETMDKAREFCKKNFGDLVSIQS  840
             KGQTPKPEPTPAPQDNPPVTEDGWVIYKDYQYYFSKEKETMD AR FCK+NFGDLVSIQS
Human  781   KGQTPKPEPTPAPQDNPPVTEDGWVIYKDYQYYFSKEKETMDNARAFCKRNFGDLVSIQS  840

Pig    841   ESEKKFLWKYVNKNDAQPAYFIGLLISLDKKFIWMDGSKVDYVAWAAGEPNFANDDENCV  900
             ESEKKFLWKYVN+NDAQ AYFIGLLISLDKKF WMDGSKVDYV+WA GEPNFAN+DENCV
Human  841   ESEKKFLWKYVNRNDAQSAYFIGLLISLDKKFAWMDGSKVDYVSWATGEPNFANEDENCV  900

Pig    901   TMYTHSGFWNDINCGYPNAFICQRHNSSINA-TVTPTIPSAPGGCKEGWNFYNDKCFKIF  959
             TMY++SGFWNDINCGYPNAFICQRHNSSINA TV PT+PS P GCKEGWNFY++KCFKIF
Human  901   TMYSNSGFWNDINCGYPNAFICQRHNSSINATTVMPTMPSVPSGCKEGWNFYSNKCFKIF  960

Pig    960   GFVEEERKNWQEARKACIGFGGNLASIRNEKEQAFLTYHMKDSTFNAWTGLNDVNSEHTF  1019
             GF+EEERKNWQEARKACIGFGGNL SI+NEKEQAFLTYHMKDSTF+AWTGLNDVNSEHTF
Human  961   GFMEEERKNWQEARKACIGFGGNLVSIQNEKEQAFLTYHMKDSTFSAWTGLNDVNSEHTF  1020

Pig    1020  LWTDGRGVHYTNWGKGYPGGRRSSLSYEDADCVVIIGGKSRDAGKWMDDICDNKRGYICQ  1079
             LWTDGRGVHYTNWGKGYPGGRRSSLSYEDADCVVIIGG S +AGKWMDD CD+KRGYICQ
Human  1021  LWTDGRGVHYTNWGKGYPGGRRSSLSYEDADCVVIIGGASNEAGKWMDDTCDSKRGYICQ  1080

Pig    1080  TLPDSSLPRSPTTIPTDGFIKYGESSYSLTKLKLQWHEAADYCKLHSSLIASILDPYSNA  1139
             T  D SL   P TI TDGF+KYG+SSYSL + K QWHEA  YCKLH+SLIASILDPYSNA
Human  1081  TRSDPSLTNPPATIQTDGFVKYGKSSYSLMRQKFQWHEAETYCKLHNSLIASILDPYSNA  1140

Pig    1140  FAWMQMQALSEPVWIALNSNLTNNEYVWTDKWRVRYTNWAADEPRLKTACVYMDLDGSWK  1199
             FAW+QM+  +E VWIALNSNLT+N+Y WTDKWRVRYTNWAADEP+LK+ACVY+DLDG WK
Human  1141  FAWLQMETSNERVWIALNSNLTDNQYTWTDKWRVRYTNWAADEPKLKSACVYLDLDGYWK  1200

Pig    1200  TANCNESFYFFCKKSDETPATEPPQLPGRCPESEHTAWIPFHGHCYYIESSYTRNWGQAS  1259
             TA+CNESFYF CK+SDE PATEPPQLPGRCPES+HTAWIPFHGHCYYIESSYTRNWGQAS
Human  1201  TAHCNESFYFLCKRSDEIPATEPPQLPGRCPESDHTAWIPFHGHCYYIESSYTRNWGQAS  1260

Pig    1260  LECLRMGSSLVSIESAAESSFLSYRVEPLQSKTNFWIGLYRNVEGMWLWVNNNPVSFVNW  1319
             LECLRMGSSLVSIESAAESSFLSYRVEPL+SKTNFWIGL+RNVEG WLW+NN+PVSFVNW
Human  1261  LECLRMGSSLVSIESAAESSFLSYRVEPLKSKTNFWIGLFRNVEGTWLWINNSPVSFVNW  1320
```

```
Pig    1320  NTGDPSGERNDCVALYASSGFWNNIHCSSYKGYICKRPKIVDAEPTHALVTTKADPRKMV  1379
             NTGDPSGERNDCVAL+ASSGFW+NIHCSSYKGYICKRPKI+DA+PTH L+TTKAD RKM
Human  1321  NTGDPSGERNDCVALHASSGFWSNIHCSSYKGYICKRPKIIDAKPTHELLTTKADTRKMD  1380

Pig    1380  TSKPSSNSAGVVVIVVLLILTGAGFAAYFFYKKRRVHLPQEENFENTLYFNSASTPGASD  1439
              SKPSSN AGVV+IV+LLILTGAG AAYFFYKKRRVHLPQE  FENTLYFNS S+PG SD
Human  1381  PSKPSSNVAGVVIIVILLILTGAGLAAYFFYKKRRVHLPQEGAFENTLYFNSQSSPGTSD  1440

Pig    1440  TKDLIGNIEQNEHVVI  1455
              KDL+GNIEQNEH VI
Human  1441  MKDLVGNIEQNEHSVI  1456
```

Anti-CD206 (1) (1400-1441 aa).

Anti-CD206 (2) (1090-1389 aa).

Anti-CD206 (3) (C-terminal).

Figure S2. Homology sequence analysis of pig and human CD206 proteins. Blue color shows the peptide region used for immunization to obtain Anti-CD206 (1) antibody. Red color shows the peptide region used for immunization to obtain Anti-CD206 (2) antibody.
