# Supplementary material for: Non-Specific Binding, a Limitation of the Immunofluorescence Method to Study Macrophages In Situ
Source: Genes (Basel). 2021 Apr 27;12(5):649. doi: 10.3390/genes12050649 (PMC8145419; doi:10.3390/genes12050649)
Supplement: Supplementary file 1 [file genes-12-00649-s001.zip › FigureS3new.pdf]

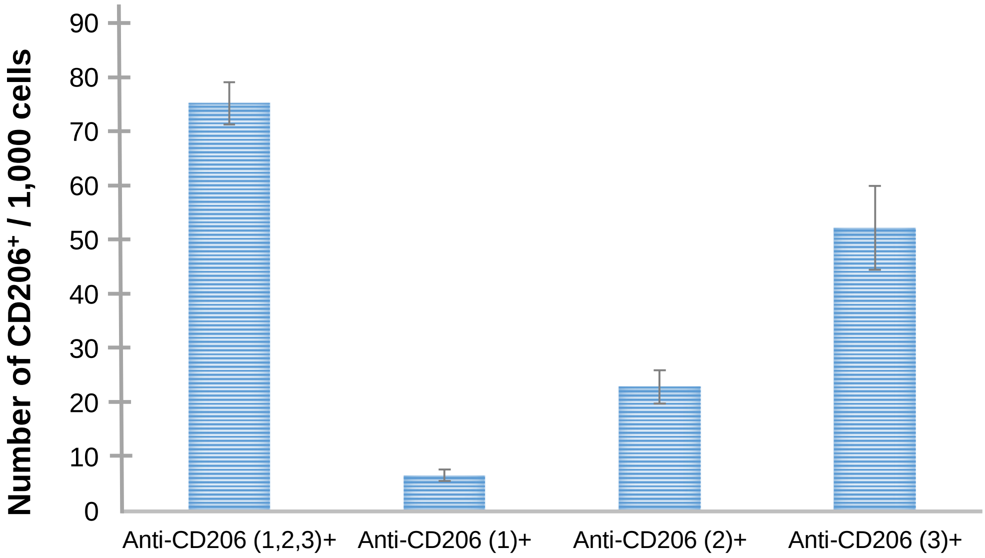

Figure S3. Quantitative analysis of the CD206 expressing cells, based on random examination of 3 sets of 1,000 cells per condition.
