# Supplementary material for: Non-Specific Binding, a Limitation of the Immunofluorescence Method to Study Macrophages In Situ
Source: Genes (Basel). 2021 Apr 27;12(5):649. doi: 10.3390/genes12050649 (PMC8145419; doi:10.3390/genes12050649)
Supplement: Supplementary file 1 [file genes-12-00649-s001.zip › TableS1.pdf]

Oligo name	Gene accession number	Oligo sequence
CD206_DIG_FW_T3	NM_001255969	TGAATTAACCCTCACTAAAGGGATGGTCACTTCCAAGCCTTCTTC
CD206_DIG_RV_T7	NM_001255969	TGTAATACGACTCACTATAGGGCAAACAGGACAAAGGACACATCTGC
I1B2_CD206_HCR1_I2B2	NM_001255969	CCTCGTAAATCCTCATCAATCATCCAGTAAACCGCCAAAAAATTGTATATCAAAAACTGCCTAGTGTCCAAGAGCTGCAGGGCAACCGGAAGAAAAAAAGCTCAGTCCATCCTCGTAAATCCTCATCAATCATC
I1B2_CD206_HCR2_I2B2	NM_001255969	CCTCGTAAATCCTCATCAATCATCCAGTAAACCGCCAAAAAAAGTTGCCCAAGAGCGTGTACATGGCTTCATAACCTCGAGAGCATAGATCAAAAAAAAGCTCAGTCCATCCTCGTAAATCCTCATCAATCATC
I1B2_CD206_HCR3_I2B2	NM_001255969	CCTCGTAAATCCTCATCAATCATCCAGTAAACCGCCAAAAAATCCATAACCTCTCGATGCCCTCAAATTTCAATGGACAATATCCAAATAGCAAAAAAAGCTCAGTCCATCCTCGTAAATCCTCATCAATCATC
I1B2_CD206_HCR4_I2B2	NM_001255969	CCTCGTAAATCCTCATCAATCATCCAGTAAACCGCCAAAAAATCCTGAGGTCAAGGAACTGGTTAATCCTGTCAGGTACGTTTGCTCGTGGAAAAAAAAGCTCAGTCCATCCTCGTAAATCCTCATCAATCATC
I1B2_CD206_HCR5_I2B2	NM_001255969	CCTCGTAAATCCTCATCAATCATCCAGTAAACCGCCAAAAAATTTCCGGGTTCTGCTGATGGACTTCCTGGTAGCCAGTTCAAATATCGGAAAAAAAAAGCTCAGTCCATCCTCGTAAATCCTCATCAATCATC

Table S1. Oligos used for *in situ* hybridization experiments. T3 (T3 promoter), T7 (T7 promoter), FW (forward primer), RV (reverse primer).
