# Supplementary figures and images for: Non-Specific Binding, a Limitation of the Immunofluorescence Method to Study Macrophages In Situ
Source: Genes (Basel). 2021 Apr 27;12(5):649. doi: 10.3390/genes12050649 (PMC8145419; doi:10.3390/genes12050649)

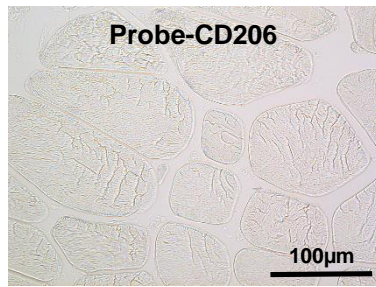

**Figure S4.** Expression of CD206 mRNA in the non-treated muscle by *in situ*-DIG hybridization.

Supplement: Supplementary file 1 [file genes-12-00649-s001.zip › FigureS4new.pdf]
